# Case report: dual primary malignancies treated by laparoscopic multiorgan resection with natural orifice specimen extraction surgery

**DOI:** 10.3389/fonc.2022.916104

**Published:** 2022-07-29

**Authors:** Kunpeng Hu, Yifan Ke, Qin Chen, Jiezhong Wu, Yingping Ke, Qiuxian Xie, Bo Liu, Jiajia Chen

**Affiliations:** ^1^ Department of General Surgery, The Third Affiliated Hospital of Sun Yat-Sen University, Guangzhou, China; ^2^ Department of General Surgery, Chaozhou Central Hospital, Chaozhou, China; ^3^ Department of Gynecology, Chaozhou Central Hospital, Chaozhou, China

**Keywords:** natural orifice specimen extraction surgery (NOSES), dual primary malignancies, laparoscopic multiorgan resection, intrahepatic cholangiocarcinoma (ICC), endometrial cancer, case report

## Abstract

With microtrauma becoming a consensus, in order to improve surgical treatment capability, the clinical application of laparoscopic multiorgan resection is becoming more and more complicated and diversified. Recently, we successfully presented a case of transvaginal specimen extraction surgery that included laparoscopic anatomical left hemihepatectomy combined with laparoscopic total hysterectomy and bilateral adnexectomy and the pelvic and para-aortic lymphadenectomy. The patient, a 75-year-old woman, was hospitalized with abnormal vaginal discharge and bleeding. The pathologic diagnosis of uterine curettage was endometrioid adenocarcinoma. After completing examinations such as color Doppler ultrasound, CEUS, MRCP and thoracoabdominal enhanced spiral CT, preoperative diagnosis was considered as endometrial cancer and a space-occupying lesion in the liver (primary or secondary site)?. No lymphatic or distant metastasis had been found. We also excluded Lynch syndrome by digestive endoscopy and gene sequencing. After a multidisciplinary consultation, the patient underwent surgery under general anesthesia on 24 September 2021. The operation was completed uneventfully in 6 hours, then the patient was transferred to the ICU for follow-up monitoring. The patient began to eat and was able to leave bed on the 4^th^ postoperative day. According to immunohistochemistry, the patient’s postoperative diagnosis was intrahepatic cholangiocarcinoma (ICC) and endometrial cancer. Compared with open surgery, laparoscopic multiorgan resection with natural orifice specimen extraction surgery (NOSES) has many advantages such as fewer traumas, shorter recovery time, and better postoperative quality of life. However, combined large-scale laparoscopic surgeries of different organs can be challenging for surgeons and anesthesiologists. No similar cases have been searched.

## Introduction

Endometrial cancer is a type of endometrioid adenocarcinoma that is common in perimenopausal women. In developed countries, endometrial cancer is the most common gynecologic malignancy. The main symptoms are abnormal vaginal bleeding and discharge. Tumor metastasis includes hematogenous dissemination, lymphatic system invasion and direct invasion. Laparoscopic total hysterectomy and bilateral adnexectomy combined with pelvic and para-aortic lymphadenectomy is the standard treatment for endometrial cancer (1).

ICC is a type of adenocarcinoma that originates in the epithelium of secondary bile ducts and its branches. ICC accounts for approximately 10%-15% of primary malignancies in the liver, and the incidence rate has increased in recent years. ICC lacks characteristic symptoms, so its early diagnosis and long-term prognosis are poor. Radical resection is the main treatment for ICC (2).

Although laparoscopic multiorgan resection is challenging, it avoids from the need for repeated surgery and represents a high-level surgical technique. Anatomical hepatectomy refers to the precise removal of the malignancy and the hepatic segments in which it is located anatomically. Compared with nonanatomical hepatectomy, it has advantages in terms of the incidence of postoperative complications and disease-free survival (DFS) (3). However, the prognosis of patients after anatomical hepatectomy also depends on some risk factors such as preoperative cirrhosis and tumor characteristics, so anatomical hepatectomy should be presented as an option for only eligible patients (4). In natural orifice specimen extraction surgery (NOSES), the surgeon does not need to extract specimens by enlarging the incision during laparoscopic surgery; instead, the specimen is extracted through the rectum, anal tube, or vagina. NOSES reduces patient pain and shortens recovery time. Transvaginal specimen extraction surgery is common in gynecological or colorectal surgery (5).

This case report has been reported in line with the SCARE Criteria (6).

## Case description

The patient, a 75-year-old woman who comes from Chaozhou, Guangdong, was hospitalized in the Department of Gynecology due to 4 months of abnormal vaginal discharge and 10 days of vaginal bleeding. The patient didn’t have any other symptoms. Clinicians didn’t find any positive signs on physical examination. And the patient had never received any previous diagnostic examination or treatment. She had hypertension and type 2 diabetes. Both blood pressure and glucose were stably controlled by taking Amlodipine Besylate and Metformin hydrochloride Po Qd. The patient also denied the history of surgery, trauma, blood transfusion, drug allergy, smoking and drinking. Her family members were all healthy, without history of cancer or genetic disease.

The pathologic result of diagnostic uterine curettage was endometrioid adenocarcinoma. However, abdominal color Doppler ultrasound revealed a low echo-level focus in segment IV of the liver with intrahepatic cholangiectasis in the left hepatic lobe. In addition, there were no significant abnormalities in the biliary system, pancreas, spleen, urinary system or double annexa.

To further determine the properties of the liver focus, we also performed MRCP and contrast-enhanced ultrasound (CEUS) in the liver. CEUS indicated that the low echo-level focus was ICC. MRCP revealed a space-occupying lesion that encroached on the hepatic portal and left lobe. To determine the stage of the tumor, we performed thoracoabdominal enhanced spiral CT, and the results were as follows: 1. ICC in segment IV with intrahepatic cholangiectasis, 2. endometrial cancer, 3. chronic inflammation and a nodule in the inferior lobe of the left lung, 4. an increscent lymph node in the mediastinum, and 5. no space-occupying lesions in the extrahepatic biliary system, pancreas, spleen or urinary system.

Since the patient was thought to have two primary malignancies and approximately 50% of women with Lynch syndrome have clinical manifestations of endometrial cancer as initial symptoms (7), she was transferred to the department of general surgery for follow-up diagnosis and treatment. Then, we performed digestive endoscopy to eliminate Lynch syndrome, and the result was chronic superficial gastritis. The patient’s serum ferritin was 458.70 µg/L, and her CA199 was 45.15 ku/L. Other serum tumor markers were in the normal range.

In summary, the preoperative diagnosis comprised a space-occupying lesion in the liver, endometrial cancer, hysteromyoma, hypertension and type-2 diabetes. The space-occupying lesion in the liver may be ICC, hepatocellular carcinoma or metastatic carcinoma. We intended to confirm the diagnosis by postoperative pathological examination. No lymphatic or distant metastasis had been found. According to the results of auxiliary examination, the patient’s surgeons and gynecologists developed a protocol for transvaginal specimen extraction surgery (NOSES): laparoscopic anatomical left hemihepatectomy combined with laparoscopic total hysterectomy and bilateral adnexectomy and pelvic and para-aortic lymphadenectomy.

Due to the risk of large-scale surgery in older patients, we performed exhaustive preoperative examinations. Fortunately, in addition to slight hypopnea, the functions of other systems and the vital signs were normal. The result of Holter monitor ECG was sinus rhythm with several ventricular premature beats and atrial premature beats. There were no significant abnormalities on cardiac color Doppler ultrasound, spiral CT of the coronary artery or lung function examination. After multidisciplinary consultation and a complete preoperative evaluation, the surgical protocol was considered feasible.

**Figure 1:**
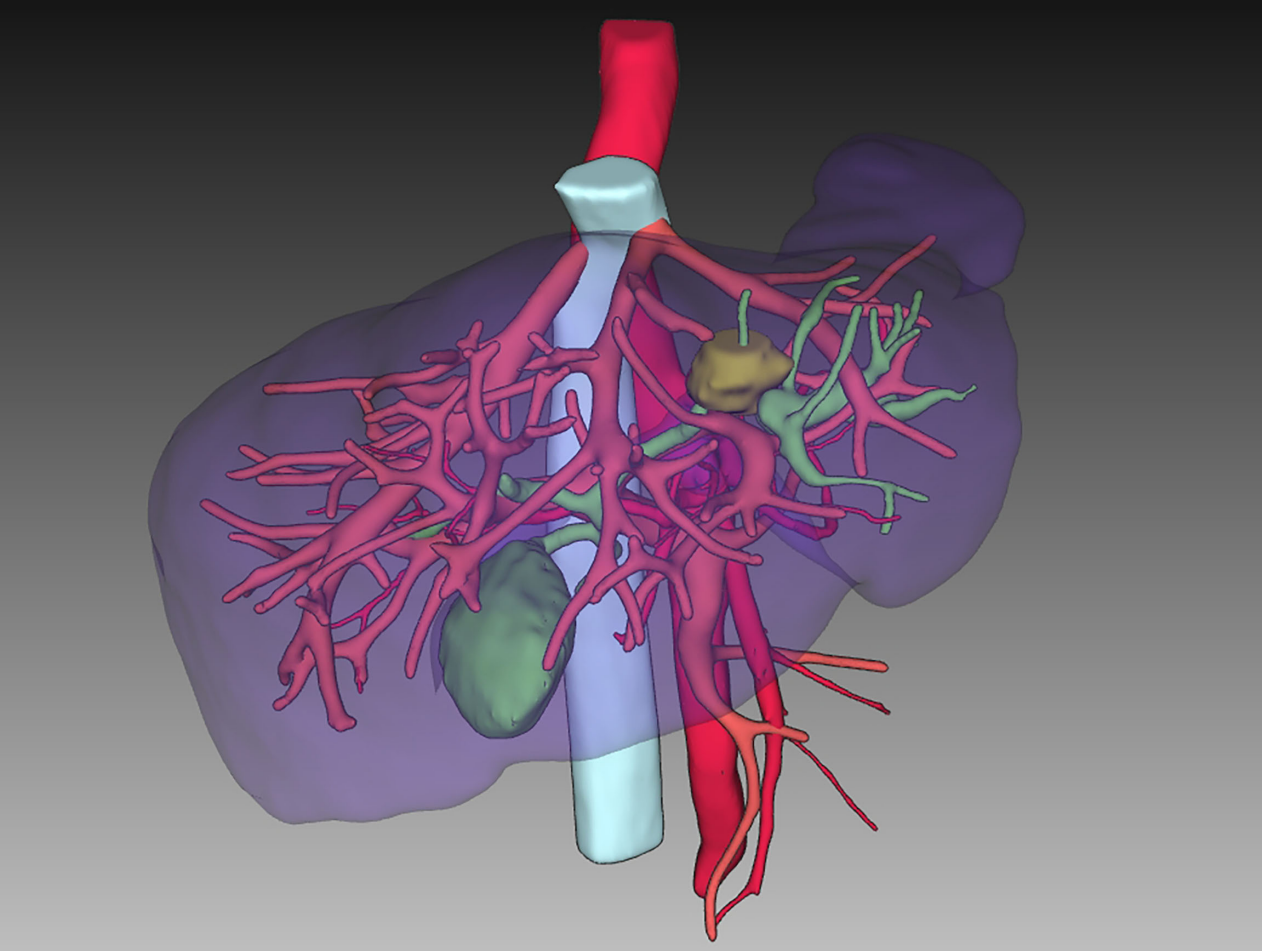
The preoperative 3D-restruction of liver.

**Figure 2:**
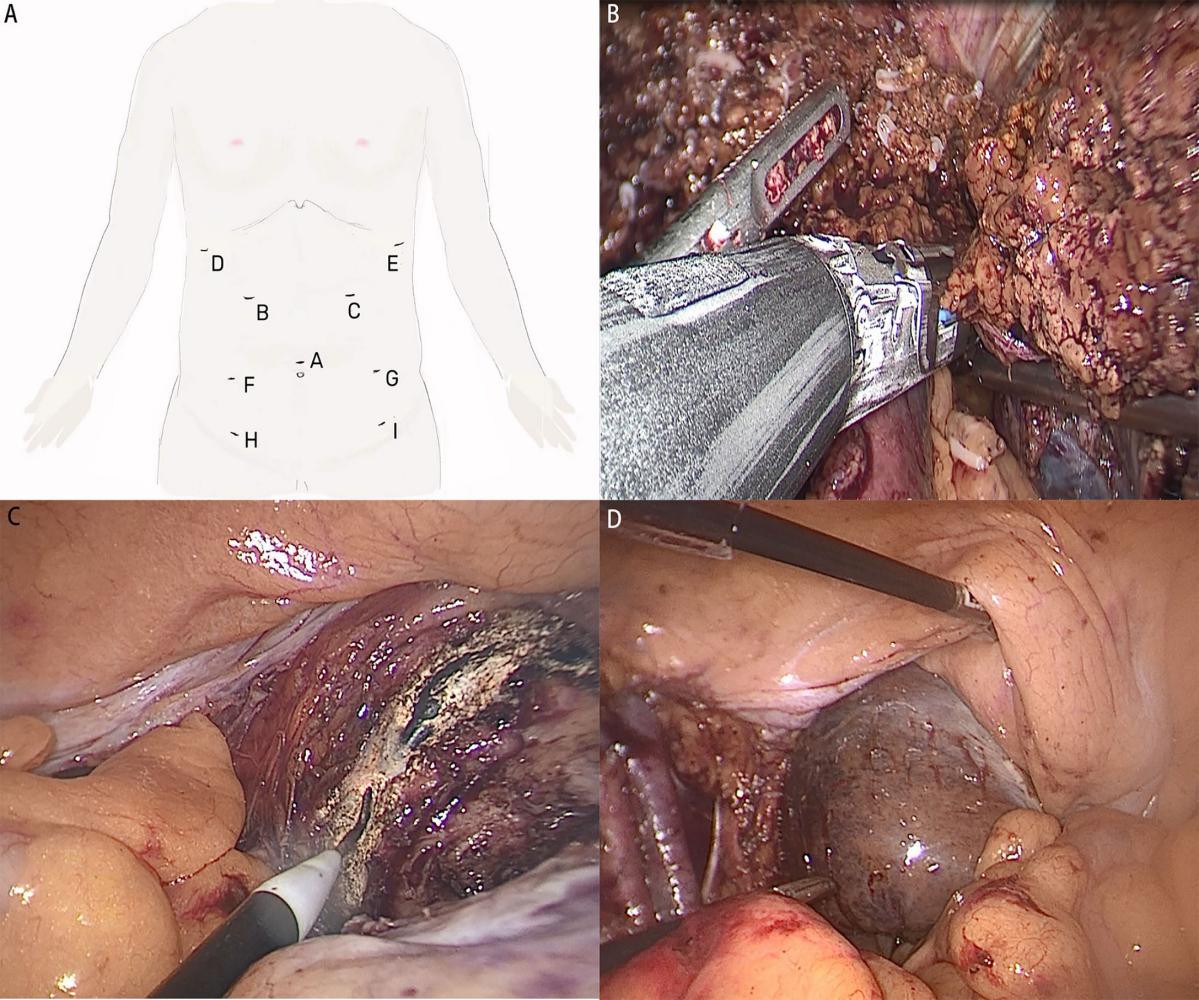
**(A)** Trocars during the operation. **(B)** Resect the left hepatic vein and lobe. **(C)** Cut off the vaginal wall and extract uterus. **(D)** Extract liver specimens through the vagina.

**Figure 3:**
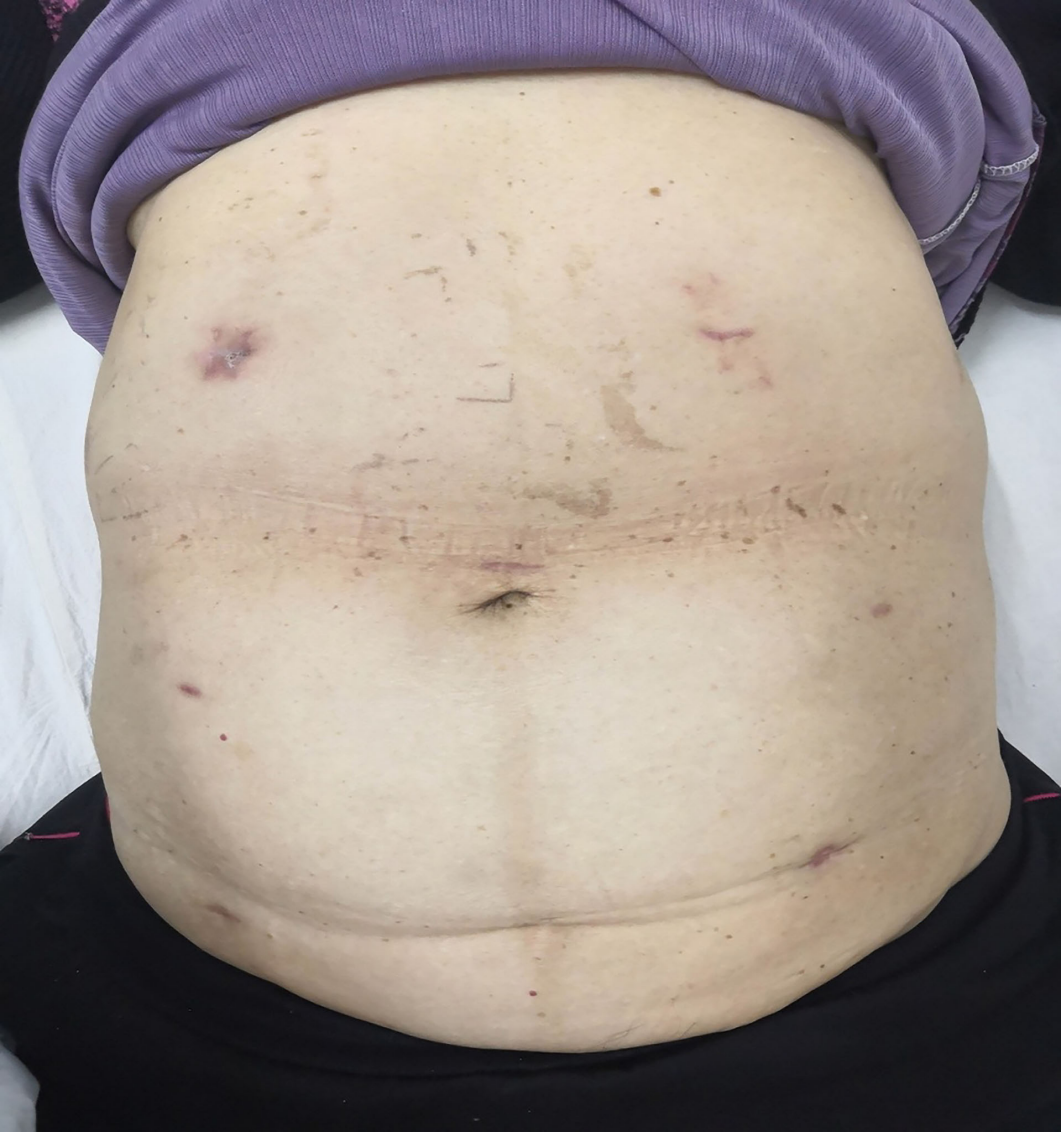
The patient’s abdomen after dermal sutures out.

After adequate preoperative communication and preparation such as abrosia, enema, cross-matching blood test and prophylactic antibiotics, the patient underwent surgery under general anesthesia on 24 September 2021 in Chaozhou central hospital. The chief surgeon and gynecologist were all experienced with more than 15 years of career. The patient was placed in the reverse Trendelenburg’s position. All the surgical procedures were performed under laparoscopy. Thin-layer CT-Scan and 3D digital reconstruction guided the surgeons in identifying the anatomic structures of organs during laparoscopic microtrauma surgery (**Figure 1**), thus accurately determining the excisional range and preserving normal liver tissue as much as possible while achieving a radical cure. With the help of an anesthesiologist who precisely maintained a low central venous pressure, surgeons performed laparoscopic anatomical left hemihepatectomy with little bleeding (8). Subsequently, gynecologists added four trocars and completed the laparoscopic total hysterectomy and bilateral adnexectomy and dissected the pelvic and para-aortic lymph nodes (**Figure 2**). Finally, the surgeons extracted the surgical specimens completely through the vagina, so that there were only some small stomas in the abdominal wall and no operative incision (**Figure 3**). The operation was completed uneventfully in 6 hours. The total blood loss was estimated to be 200 ml. The abdominal surgical dressings were dry and clean, without staxis or seepage. The drainage tubes for the hepatorenal recess and the hepatic incisal surface were smooth, and a dark red liquid was discharged. The drainage tube in the pelvic cavity was also smooth, and a reddish liquid was discharged.

**Figure 4:**
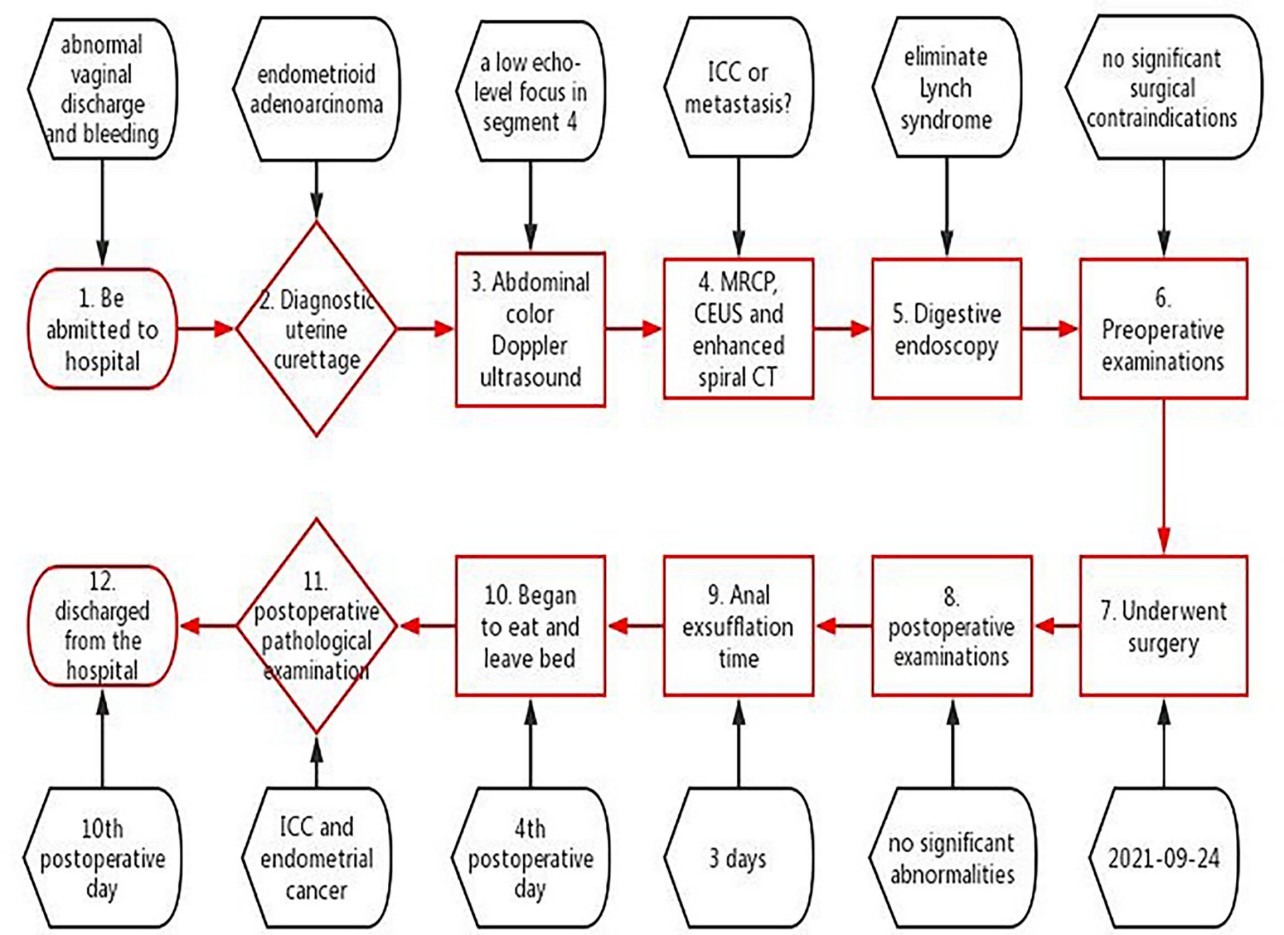
Information of care organized as a timeline.

After the operation, taking into account the patient’s risk factors, such as age, extensive surgery and a long operative time, the patient was transferred to the ICU for follow-up monitoring. The patient received oxygen therapy, fluid replacement, nutritional support, antibiotics, analgesia, acid inhibitor, liver protection and eliminate sputum treatment. The postoperative anal exsufflation time was 3 days. The patient began to eat and was able to leave bed on the 4th postoperative day. Her P/F was low after the surgery. Clinicians considered the cause may be old age, a long operative time and preoperative slight hypopnea. Therefore, the patient’s tracheal cannula was removed on the 4th postoperative day. The results of postoperative spiral CT and blood work showed no significant abnormalities. The recovery was uneventful, and the patient was discharged from the hospital on the 10th postoperative day (**Figure 4**).

Specimens included fragmented hepatic tissue (approximately 18 cm in diameter), gallbladder, complete uterus (approximately 8*7*5 cm), bilateral adnexas and lymph nodes. An incanus area (approximately 3 cm in diameter) could be seen near the hepatic portal of the ligament teres hepatis, with a tough texture and an unclear boundary. The mass (approximately 4 cm in diameter) in the uterine cavity had infiltrated the superficial muscular layer (depth<1/2). Neither tumor was accompanied by lymph node metastasis or nerve and vascular encroachment. According to the results of postoperative pathological examination and immunohistochemistry, the patient’s postoperative diagnosis was ICC (pT1aN0M0, stage IA) and endometrial cancer (pT1aN0M0, stage IA). In conclusion, the patient’s long-term prognosis is good, and the attained clinical outcomes have achieved the expectations.

Clinicians have followed up the patient since she was discharged from the hospital. The patient’s adherence and tolerance are good. She can cooperate with clinicians in postoperative follow-up, and follow our advice on treatment, lifestyle and diet. We perform abdominal spiral CT scan and blood tests including serum tumor markers and the liver function to the patient every 3 months. The results are normal, and the double primary malignancies have not recurred so far. Besides, although the patient underwent extensive surgery, she has never suffered any postoperative complications or adverse events.

## Discussion

Old age is one of the predisposing factors for malignancy. Radical resection is the primary treatment for most cancers, but such surgeries are usually extensive and cause great trauma. However, due to the aging of the body, which is prone to a variety of underlying diseases, older patients often have less tolerance for surgery (9). Although many of them have surgical indications, they are unable to receive surgery because of their poor physical condition and have to opt for nonradical therapies. Therefore, ensuring a radical cure while minimizing trauma is always a problem for the surgical treatment of cancers.

Recently, due to advances in minimally invasive surgical technology and perioperative support treatment, some surgical contraindications are no longer a problem. However, some intractable cases, such as malignancy with distant metastasis or multiple primary cancers *in vivo*, remain a major challenge for surgeons (10). For example, a conventional laparotomy may be accompanied by with enormous trauma and high mortality. A series of asynchronous laparoscopic resections not only lengthen the treatment cycle but also cause the patient to suffer unnecessary pain, so it is not worth the risk. In contrast, although simultaneous laparoscopic multiorgan resection is more difficult and has a higher risk of conversion to laparotomy, this is still the best choice if appropriate techniques and equipment are used (11, 12).

NOSES is a new technology that supports the trend of minimally invasive surgery. This means that surgeons do not need to extract specimens by enlarging incisions during the course of laparoscopic surgery. NOSES is common in gynecological and colorectal surgery and has extensive applications in other surgical fields (13, 14). Although there was a case of laparoscopic hepatectomy with transvaginal specimen extraction surgery in 2008 (15), no case of NOSES combined with laparoscopic hepatectomy and total hysterectomy and bilateral adnexectomy has been reported. NOSES prevents postoperative and incision-related complications, reduces postoperative pain, and achieves better abdominal cosmetic results (16). However, all NOSES procedures are performed under laparoscopy, and there are potential risk factors, such as peritoneal infection and tumor cell peritoneal seeding (17). Therefore, NOSES requires excellent surgical techniques and a long operative time.

Multiple primary malignancies are often considered to be caused by a hereditary neoplastic syndrome (e.g., Lynch syndrome) (18). In addition, other risk factors (e.g., old age, tobacco, alcohol, work environment, and genetic mutations) can also make cancer patients susceptible to a synchronous or metachronous second primary malignancy (19). In this case, an elderly patient synchronously suffered from endometrial cancer and ICC. Lynch syndrome was ruled out through digestive endoscopy and gene sequencing. We found reports of ICC (20) or endometrial cancer (18) with colorectal cancer, but ICC with endometrial cancer has not been reported. Although multiple primary malignancies are rare, the prognosis is poor, and the incidence is gradually increasing (21). Therefore, timely and accurate diagnosis is essential for the treatment and prognosis of such patients. They often receive chemical or targeted therapy (22), but surgical treatment has the advantage of a radical cure, so early multiple primary malignancies should be resected *via* surgery (23). In this case, we developed a complicated and unprecedented NOSES protocol that included high-level surgeries in different departments. In 2015, surgeons performed combined Da Vinci robot-assisted laparoscopic left hepatectomy and total hysterectomy in India (24). In contrast, we creatively chose transvaginal specimen extraction surgery to minimize trauma while ensuring a radical cure.

Microtrauma and radical cure are the two key words for the surgical treatment of cancers, and anatomical hepatectomy and NOSES epitomize these two points. The challenges and benefits of surgery should both be considered. Such complicated surgeries can radically cure refractory malignancy and lower the surgical threshold for older and infirm patients. For this purpose, high-quality hospitals with skilled surgeons and advanced equipment should enable these complicated operations for the benefit of patients. However, such complicated operations are inappropriate for promotion in primary hospitals as this will increase the incidence of surgical complications and adverse events.

In summary, the success of this case shows that the surgical protocols for patients with refractory malignancies should be elaborate and personalized. In cases of refractory malignancy, surgeons should consider the difficulty and risk of surgery, and do all they can to achieve a radical cure while ensuring patients’ postoperative quality of life to maximize patient benefits.

## The patient’s perspective

I’m very grateful to Dr. Hu and his team for their loving care. Though I had the option to receive radical surgery, cancer and surgery both frightened me. Fortunately, the surgical protocol designed by Dr. Hu’s team was satisfactory. It was a minimally invasive surgery, which means they didn’t have to make a huge incision on my abdominal wall. And I needn’t undergo a revision surgery. So, I received surgery on 24 September 2021. It was completed successfully. Postoperative recovery was uneventful. No complications or cancer recurrence has occurred so far. I’m satisfied with my postoperative quality of life.

## Data availability statement

The original contributions presented in this case report are included in the article/supplementary material. Further inquiries can be directed to the corresponding authors.

## Ethics statement

Written informed consent was obtained from the individual for the publication of any potentially identifiable images or data included in this article

## Author contributions

KH: protocol development, surgical operator and manuscript revision. YFK: data collection and manuscript writing. QC: surgical assistant and clinical follow-up. JW: surgical assistant. YPK: clinical follow-up. QX: operator of gynecological surgery. BL: surgical direction. JC: project assistant and imaging data collection. All authors contributed to the article and approved the submitted version.

## Funding

Natural Science Foundation of Guangdong Province of China (2021A1515012493). Scientific Research Project of Dongguan Binhaiwan Central Hospital (2021001).

## Acknowledgments

The authors thanks Dr. Zhen Xu for his help in this article.

## Conflict of interest

The authors declare that the research was conducted in the absence of any commercial or financial relationships that could be construed as a potential conflict of interest.

## Publisher’s note

All claims expressed in this article are solely those of the authors and do not necessarily represent those of their affiliated organizations, or those of the publisher, the editors and the reviewers. Any product that may be evaluated in this article, or claim that may be made by its manufacturer, is not guaranteed or endorsed by the publisher.
