# Corrigendum: Case report: dual primary malignancies treated by laparoscopic multiorgan resection with natural orifice specimen extraction surgery

**DOI:** 10.3389/fonc.2023.1297241

**Published:** 2023-10-19

**Authors:** Kunpeng Hu, Yifan Ke, Qin Chen, Jiezhong Wu, Yingping Ke, Qiuxian Xie, Bo Liu, Jiajia Chen

**Affiliations:** ^1^ Department of General Surgery, The Third Affiliated Hospital of Sun Yat-Sen University, Guangzhou, China; ^2^ Department of General Surgery, Chaozhou Central Hospital, Chaozhou, China; ^3^ Department of Gynecology, Chaozhou Central Hospital, Chaozhou, China

**Keywords:** natural orifice specimen extraction surgery (NOSES), dual primary malignancies, laparoscopic multiorgan resection, intrahepatic cholangiocarcinoma (ICC), endometrial cancer, case report

Incorrect Funding

In the published article, there was an error in the Funding statement. The funding statement for the Natural Science Foundation of Guangdong Province of China was displayed as “2021A15150124”. The correct statement is “Natural Science Foundation of Guangdong Province of China (2021A1515012493).’’ The correct Funding statement appears below.

Funding

Natural Science Foundation of Guangdong Province of China (2021A1515012493).

Scientific Research Project of Dongguan Binhaiwan Central Hospital (2021001).

The authors apologize for this error and state that this does not change the scientific conclusions of the article in any way. The original article has been updated.

